# Chagas disease treatment: a 120-year-old challenge to public health

**DOI:** 10.1590/0074-02760210501chgsa

**Published:** 2022-05-23

**Authors:** Samuel Goldenberg

**Affiliations:** 1 Fundação Oswaldo Cruz-Fiocruz

Chagas disease (CD), also known as American trypanosomiasis, results in more than 8,000 deaths per year. Reports of new cases of CD through vector transmission have decreased due to vector control policies, improvements in the quality of life of people living in rural regions, and compulsory blood screening in several countries.

Nevertheless, there is a contingent of more than 6 million individuals who remain infected with *Trypanosoma cruzi*, which require medical assistance and lifelong treatment, and this number may increase due to impacts on public health policies resulting from the Coronavirus disease 19 (COVID-19) pandemic. To date, there is no perspective on an efficacious vaccine against trypanosomiasis, and the alternative is the development of safe and efficient chemotherapies to treat this disease.

The drugs currently available for treating CD, nifurtimox and benznidazole, were introduced more than 50 years ago. However, the effectiveness of nifurtimox and benznidazole is restricted to the initial phases of the disease, they are not suitable for the treatment of chronic cases and they can impart various collateral effects. Other formulations (e.g., posaconazole, fexinidazole and inhibitors of ergosterol synthesis) or combinations of benznidazole with other compounds have been tested but have worse performances than benznidazole, the typical treatment. Hence, the discovery of new drugs or drug repositioning is a top priority.

Recent technological achievements, such as high content screening, associated with the unveiling of the functional genomes of trypanosomes, should contribute to the detection and validation of new targets for CD chemotherapy. An ideal drug would be effective against all strains and distinct typing units (DTUs) of the parasite and could be employed to treat all clinical forms of the disease. This is an important requirement to avoid the failure of the treatment due to strain resistance, as observed for the drugs commonly used for CD prophylaxis.

It is surprising that although *T. cruzi* presents several special features compared to other eukaryotic organisms, such as mRNA polycistronic transcription, glycosomes, kinetoplasts with a network of maxi- and minicircles, and extensive mitochondrial RNA editing, it is a challenge to develop specific drugs acting on the functioning enzymes that confer uniqueness to trypanosomatids in nature.

It is possible that treatment failure in CD may be due to the existence of dormant forms (persisters) of *T. cruzi* that persist after acute infection. Accordingly, it is not clear in which tissues/cells the parasites hide in chronic phase patients and what, other than immune failure, determines the evolution to a chronic symptomatic form of disease.

The absence of an animal model that mimics the complexity of the disease has impaired progress in the study of CD, despite the claims from several authors that postulate the reliability of their models.

Nonetheless, progress in parasite-host interactions should increase due to the improvement of high-precision bioluminescence imaging, which allows the visualization of *T. cruzi* during infection in animal models after parasite inoculation. In addition, new genetic manipulation tools, such as CRISPR-Cas technology, would allow the manipulation of target genes to obtain further insight into parasite-host interactions.

The actual strategies for CD prophylaxis are mainly focused on targeting the parasite. Additional efforts should be invested in addressing the treatment of CD to address issues related to human host responses, particularly the modulation of the host immune response, hence allowing a broader understanding of the complex pathophysiology of CD.

Accordingly, the prognostic of the disease would be a remarkable breakthrough because preventive measures could be implemented to avoid or diminish damage to patients.

The accompanying article by Kratz et al. provides a fully referenced comprehensive review of some of the topics discussed above. Carlos Chagas first described the disease 120 years ago, which was a remarkable scientific contribution. Despite the numerous studies performed since that description, we still face the challenge of eradicating this important neglected disease.
